# The association of *RNF34* and *RNF128* with carcass and meat quality traits of Chinese Simmental-cross steers

**DOI:** 10.5194/aab-68-299-2025

**Published:** 2025-05-20

**Authors:** Jun-Zheng Zhang, Azher Nawaz, Si-Han Wang, Quan Tian, Chun-Yin Geng, Ying Hai-Jin, Shuang Ji

**Affiliations:** 1 Department of Animal Science, College of Agriculture, Yanbian University, Yanji 133000, Jilin Province, PR China; 2 Engineering Research Centre of North-East Cold Region Beef Cattle Science and Technology Innovation, Ministry of Education, Yanji 133000, Jilin Province, PR China

## Abstract

In Chinese Simmental-cross steers, carcass and meat quality traits are investigated by identifying SNPs (single-nucleotide polymorphisms) in the *RNF34 *and *RNF128 *genes. Statistical analysis showed that for 3^′^ UTR-588 G > A, a SNP of *RNF34* was significantly associated with kidney weight, testis weight, and tare weight ( P<0.05 ), and for I1-2380C > T, a SNP of *RNF128* was significantly related to forepaw weight, dressed weight, carcass brisket depth, carcass depth, and hind leg length ( P<0.05 ). This study highlights the importance of polymorphism, suggesting that *RNF34* and *RNF128* polymorphisms could be important genetic factors that affect carcass and meat quality in beef cattle. Therefore, in beef cattle production and breeding, these SNPs might be valuable marker in future marker-assisted selection programs to determine meat quality traits. However, validation in a larger sample size of the Simmental-cross steers is necessary to verify these findings across a broader population.

**Background**: This research aims to explore the potential influence of really interesting new gene (RING) finger protein (*RNF34)* 3^′^ UTR-588G > A and RING finger protein (*RNF128)* I1-2380C > T polymorphisms associated with carcass and meat quality traits in Chinese Simmental-cross steers. **Methods**: Restriction enzyme digestion and sequencing is employed to detect genotypes of *RNF34* 3^′^ UTR-588 G > A and *RNF128* I1-2380C > T in Chinese Simmental-cross steers. Then, the association of novel single-nucleotide polymorphisms in the 3^′^ UTR region of *RNF34* and the intron regions of the *RNF128* gene is analyzed to determine the meat quality traits of the animals. **Results**: This study revealed a significant association between RNF34 3^′^ UTR-588 G > A and kidney weight, testis weight, and tare weight ( P<0.05 ). Furthermore, the *RNF128* I1-2380C > T variant exhibited a significant link to multiple carcass measurements, indicating its potential association with forepaw weight, dressed weight, carcass depth, hind leg length, and carcass brisket depth ( P<0.05 ). **Conclusion**: This study highlights the importance of genetic factors that link the variations in *RNF34* and *RNF128* and their influence on carcass and meat quality in beef cattle. Thus, these variants might be novel valuable markers for meat quality traits in future marker-assisted selection programs in beef cattle breeding and production.

## 1 Introduction

With the advancements in molecular biology techniques, the identification of single-nucleotide polymorphisms (SNPs) has been widely used to study the effects of genetic mutations on animal performance, which could be used for a molecular marker-assisted approach to breeding and production (Reshma and Das, 2021; Aly and Sabri, 2015; Das et al., 2021; Deng et al., 2023; Ye et al., 2022; Nawaz et al., 2024). RING (really interesting new gene) finger proteins, *RNF34* and *RNF128* play an important role in many biological processes, such as protein degradation; protein–protein interaction; and cellular pathways such as signal transduction, cell cycle regulation, DNA repair, inflammatory response, and apoptosis (Cai et al., 2022b). Several papers showed that E3 ubiquitin-protein ligases are master regulators of adaptive thermogenesis and energy metabolism in brown fat cells. The *RNF34* is a bona fide E3 ubiquitin-protein ligase for the peroxisome proliferator-activated receptor gamma coactivator 1α (PGC- 1α ) and negatively regulates brown fat cell metabolism (Sheng et al., 2023).

*RNF34* binds to the PGC- 1α at its C-terminal region and targets it for degradation independently of the previously identified N-terminal phosphor-degron motif (Liu et al., 2023; Wei et al., 2012; Liu et al., 2008b). Deactivating *RNF34* within brown fat cells disrupts its interaction with PGC- 1α protein levels and subsequently upregulates UCP1 (uncoupling protein 1), ultimately resulting in increased oxygen consumption (Qi and Ding, 2012; Wang and Deng, 2011b). Nevertheless, the contrary effects are observed in brown fat cells with ectopic expression of wild-type *RNF34* instead of its ligase activity-defective mutant (Heissmeyer and Rao, 2004). Interestingly, β3 -adrenergic receptor signaling and cold exposure, conditions that trigger expression of PGC-1 α expression, suppress *RNF34* expression in brown fat cells. This reveals a dynamic interplay between E3 ligase for PGC-1 α , *RNF34*, and thermogenesis, suggesting its physiological importance in heat production regulation (Janowski et al., 2012; Schartner et al., 2009b).

The *RNF128* gene participates in cellular processes such as cell cycle regulation, DNA repair, inflammatory response, and apoptosis (Schartner et al., 2009b; Bai et al., 2020). The activation of the T cell is tightly regulated to avoid autoimmunity. The GRAIL protein, associated with energy metabolism in T cells, is encoded by *RNF128* and an E3 ubiquitin-protein ligase associated with T cell tolerance. Intriguingly, degradation and ubiquitination of CD40L by *RNF128* are one cause of T cell incompetence (Nurieva et al., 2010; Anandasabapathy et al., 2003). In the latest research, it has been found that the expressions of *RNF128* and *RNF34* displayed a substantial difference between younger and adult cattle (Liu et al., 2008b).

The selection of the *RNF34* and *RNF128* genes in this study was based on their functionality regarding metabolic pathways, with broader effects that have potentially indirect implications for carcass and meat quality traits in cattle. Although *RNF34* is principally associated with proteolytic degradation via its function as an E3 ubiquitin-protein ligase and *RNF128* appears to be a regulator of some immune response T cell activation genes, these quantitative trait loci (QTLs) may also influence metabolism for energy production under heat or oxidative stress conditions that affect muscle (energy content) and fat deposition in livestock (Sheng et al., 2023; Cai et al., 2022a).

Moreover, genomic studies in other species and whole-genome research often imply that genes involved in the ubiquitination pathway and inflammation response signaling pathways may have an indirect effect on growth-related meat quality (Deng et al., 2024; Leal-Gutiérrez et al., 2018). Evidence in support of this hypothesis is the identification through QTL mapping and GWAS of regions that overlap or reside near *RNF34* and *RNF128* with associations to economically relevant traits, including those in cattle and other livestock species. Although its direct functions do not suggest an apparent association, these reports together give us the reason to explore *RNF34* and/or *RNF128* as candidate genes for carcass meat quality traits. It seems that while there is substantial research related to QTL mapping, GWAS, and candidate genes for economically relevant traits, specific evidence for *RNF34* and *RNF128* in relation to carcass meat quality traits in livestock has not yet been reported in the available literature.

To further substantiate the relationship of *RNF34* and *RNF128* with beef quality traits, we scrutinize the appropriate QTL (quantitative trait locus) and GWAS (genome-wide association study) literature. QTL mapping in cattle has been used to identify various chromosomal regions correlated with carcass weight, fat deposition, and muscle development. Some of these regions overlap with the chromosomal positions of *RNF34* and *RNF128*, which found that these genes are located in regions associated with carcass and meat quality traits. The fact that *RNF34* and *RNF128* genes are located near these regions indicates that these genes may also affect meat quality, thereby appearing in the list of candidate genes for marker-assisted selection in beef cattle breeding. Additionally, comparative studies in other species, such as pigs and chickens, have shown that genes involved in the ubiquitin-proteasome pathway, such as *RNF34*, are associated with traits such as muscle fiber development and fat deposition (Antonaros et al., 2019a; Sanglard et al., 2023a). These findings suggest that *RNF34* and *RNF128* could play a similar role in cattle, potentially influencing meat quality through their effects on cellular metabolism and tissue differentiation. This study aims to generate potential evidence to incorporate marker-assisted selection into breeding strategies such as crossbreeding and pure breeding and the vital effort to preserve important genetic diversity.

Therefore, *RNF34 *and *RNF128* may be candidate genes influencing meat quality and carcass of beef cattle (Afonso et al., 2020). At present, only a limited information is available on the genetic polymorphism of *RNF34* and *RNF128* genes in cattle, and the effect of the genetic variants of these genes remains indecisive. The main objective of the study is to examine the SNPs in these two genes and finding their association with carcass and meat quality traits in Chinese Simmental-cross steers. SNPs in these genes were determined by restriction endonuclease analysis (REA) and polymerase chain reaction–restriction fragment length polymorphism (PCR-RFLP) as these are cost-effective and simple techniques which recognize the specific DNA sequence by chosen restriction enzymes that cut the DNA at specific restriction sites to genotype the known polymorphisms (Hashim and Al-Shuhaib, 2019; Antonaros et al., 2019b; Sanglard et al., 2023b).

## 2 Materials and methods

### 2.1 Experimental materials

In this study, a total of 255 Chinese Simmental-cross steers that are 28 months of age were selected from the Baolongshan cattle farm in Inner Mongolia. The selection process involved choosing 21 bulls and 2000 female cows from the offspring of Simmental cattle. However, instead of a purely random selection, the animals were chosen based on a controlled breeding program designed to maintain genetic diversity while ensuring a representative sample of the population. The breeding program involved crosses between purebred Simmental sires and dams of known pedigree, which allowed us to account for the genetic relationships among the animals. This approach minimized potential confounding effects in the genetic association analysis by reducing the impact of population structure and relatedness.

Blood samples (10 mL) from the jugular vein were collected in acid citrate dextrose (ACD), as anticoagulant. Natural samples after the treatment were kept frozen at - 80 °C (Tian et al., 2013; Xiao et al., 2016). In total, 1 mL out of all of the blood was taken for DNA extraction under the manufacturer's protocol using a DNA extraction kit (Tiangen, Beijing, China).

### 2.2 Experimental methods

#### 2.2.1 Slaughter process

The slaughtering was performed according to established standard protocols in order to safeguard the welfare of each individual and ensure a certain level of quality within data collection. To minimize stress that could as well affect meat quality traits, all the 255 Chinese Simmental-cross steers were transported under controlled conditions to the slaughter plant. Animals were allowed 24 h rest with water but no feed (to reduce gut content) on arrival at the facility. Each cow's head was secured within the dizzying box to prevent movement. A pneumatic dizzying device was aimed precisely at the intersection of the cow's two horns and the diagonal of both eyes. The device was quickly activated to render the cow unconscious. The slaughtering process started with a stunning that was done by a captive bolt pistol for fast and humane euthanasia. The animals were then exsanguinated by severing the major blood vessels in the neck, ensuring a complete and rapid bleed-out, which is critical for both animal welfare and meat quality. After exsanguination, the carcasses were hung on a rail system and moved through the slaughter line.

The carcasses were then skinned, eviscerated, and split into halves. During evisceration, the visceral organs were carefully removed and weighed as part of the trait measurements. The carcasses were washed and immediately moved into a refrigerated room set to between 0 and 4° C, where they were kept for 24 h before further processing and trait measurement. This chilling step is essential to reduce the risk of microbial contamination and to stabilize the carcass for accurate measurement of meat quality traits.

#### 2.2.2 Trait measurements

In this study, a total of 42 traits were measured, as shown in Table 1. Before trait measurements, all the carcasses were preserved in refrigerated rooms at 0 to 4 °C for 24 h. Trait were measured on the basis of cutting standards (GB/T17238-1998) for chilled and fresh beef of China (China Standard Publishing House) (Tian et al., 2013). The final weight of the body, living quality index of beef (QIB), and rib eye area (by ultrasound) were noted before slaughter. All visceral indicators were weighed after slaughter, including the spleen, large intestine, small intestine, heart, liver, kidney, and fat belly (Guo et al., 2016; Tian et al., 2013). Other carcass properties were also recorded, including the carcass weight, slaughter rate, bone weight, head weight, tare weight, hind leg circumference, fat color score, hind leg width, and carcass brisket depth. The described measurements were determined strictly according to established measurement standards (Li et al., 2017).

**Table 1:** The number of records and mean and standard errors for traits included in the association analyses.

	*RNF128*		*RNF34*	
Trait	N	Mean ± SD	N	Mean ± SD
GW (kg)	213	506.5117 ± 55.17678	255	509.5020 ± 56.54048
CW (kg)	213	268.5399 ± 34.32056	255	269.7686 ± 34.93046
DP (%)	213	52.937934 ± 1.9399901	255	52.873255 ± 2.0756458
NWB (kg)	213	19.7546 ± 3.12495	255	19.7715 ± 3.09257
HW (kg)	213	23.4677 ± 2.29767	255	23.6072 ± 2.33130
FW (kg)	213	6.0470 ± 0.69623	255	6.0660 ± 0.69769
WH (kg)	213	2.8099 ± 0.37400	255	2.8207 ± 0.37256
PW (kg)	213	39.2985 ± 4.58529	255	39.6396 ± 4.61396
LI (kg)	213	0.6104 ± 0.14412	255	0.6130 ± 0.14707
SI (kg)	213	9.1315 ± 1.05708	255	9.1348 ± 1.06245
RW (kg)	213	7.1634 ± 0.79451	255	7.1755 ± 0.78780
OW (kg)	213	3.6385 ± 0.51115	255	3.6673 ± 0.51533
HW (kg)	213	1.9730 ± 0.28008	255	1.9816 ± 0.28032
LW (kg)	213	6.5086 ± 0.68182	255	6.5322 ± 0.70268
LT (kg)	213	3.3238 ± 0.40177	255	3.3412 ± 0.39546
KW (kg)	213	1.2521 ± 0.18142	255	1.2583 ± 0.17756
SW (kg)	213	0.9036 ± 0.17818	255	0.9080 ± 0.18084
BP (kg)	213	0.3982 ± 0.06107	255	0.3996 ± 0.06006
TW (kg)	213	0.7026 ± 0.15175	255	0.7026 ± 0.15179
OT (kg)	213	1.4447 ± 0.20236	255	1.4577 ± 0.21152
MFW (kg)	213	4.8448 ± 1.07669	255	4.8787 ± 1.10583
SO (kg)	213	2.4969 ± 0.89609	255	2.5258 ± 1.10583
KFW (kg)	213	6.2017 ± 2.01893	255	6.2885 ± 2.06872
GF (kg)	213	0.8062 ± 0.36322	255	0.8000 ± 0.35013
CL (cm)	213	136.3697 ± 7.52682	255	136.7598 ± 7.39467
CD (cm)	213	63.7838 ± 3.11876	255	63.9665 ± 3.20093
CCD (cm)	213	64.5668 ± 3.46723	255	64.8087 ± 3.61591
HLC (cm)	213	49.5137 ± 3.65334	255	49.4153 ± 3.58996
HLW (cm)	213	44.6179 ± 2.70399	255	44.6279 ± 3.58996
HLL (cm)	213	78.0042 ± 2.97953	255	78.1290 ± 2.96754
TMT (cm)	213	18.1287 ± 1.76469	255	18.1342 ± 1.74472
TL (cm)	213	7.2878 ± 0.75732	255	7.3126 ± 0.76909
BFT (cm)	213	1.3142 ± 0.45328	255	1.3197 ± 0.46186
FCR (%)	213	61.7475 ± 10.52322	255	62.1734 ± 10.69373
SpH	213	5.9675 ± 0.36119	255	5.9675 ± 0.35840
PpH	213	5.4259 ± 0.29706	255	5.4124 ± 0.29315
SF (kg)	213	4.5386 ± 1.43806	255	4.5466 ± 1.44030
MBS	213	5.1556 ± 0.71330	255	5.126 ± 0.71532
EMA	213	83.7820 ± 12.18710	255	83.5498 ± 12.33113
FCC	213	5.9434 ± 0.9984	255	5.9409 ± 1.01585
FCI	213	83.3721 ± 8.02517	255	83.2598 ± 8.07216
FCS	213	2.3582 ± 0.82061	255	2.3031 ± 0.83160

GW: gross weight; CW: carcass weight; DP: dressing percentage; NWB: net weight of bone; HW: head weight; FW: forepaw weight; WH: Weight heels; LI: large intestine; SI: small intestine; RW: rumen weight; OW: omasum weight; HW: heart weight; LW: liver weight; LT: lung, trachea; KW: kidney weight; SW: spleen weight; BP: bull penis; TW: testes weight; OT: ox tail; MFW: mesenteric fat weight; SO: stomach oil; KFW: kidney fat weight; GF: genital fat; CL: carcass length; CD: carcass depth; CCD: carcass chest depth; HLC: hind leg circumference; HLW: hind leg width; HLL: hind leg length; TMT: thigh meat thickness; TL: thickness of loin; BFT: back fat thickness; FCR: fat coverage rate of carcass; SpH: slaughter pH; SF: shearing force; MBS: marbling score; EMA: eye muscle area; SE: standard error of mean.

#### 2.2.3 Ultrasound device and procedure

An Aloka SSD-500V ultrasound device with a 3.5 MHz linear array transducer was used to assess the rib eye area in live beef cattle. The transducer was inserted between the 12th and 13th rib sites, and repeated measurements were carried out to ensure accuracy. The images were analyzed using BIA Pro software, and the rib eye area was measured by detecting muscle boundaries without fat or bone structures.

#### 2.2.4 Color score assessment

The color of fat and meat was scored by adhibition equipment Olympic Minolta CR-400 chromameter. A white calibration plate (Minolta CR-A43) was used to calibrate the device prior to each session for reliable measurements (Konica Minolta Sensing, Inc., 1998). The color scores were obtained by taking readings from three different locations on the longissimus dorsi muscle for meat color and subcutaneous fat for fat color. The device provides L* (lightness), a* (redness), and b* (yellowness) values, which are then converted into a standardized color score using the CIELAB color space method.

#### 2.2.5 Primers designing and PCR amplification

Primer 5 software was used to design primers targeting bovine *RNF34* (NC_037344.1) and *RNF128* (NC_037357.1) sequences, mentioned in Table 2, while genotyping primer pairs were synthesized by Sangon Biotech (Shanghai, China). The gene-specific primer pairs are *RNF34* forward: 5^′^-CGGGCTGTTTCCCAGGTTCT-3^′^, *RNF34* reverse: 5^′^-CCCAATGATGTTGAAACGCAGA-3^′^, *RNF128* forward: 5^′^-GAGCAAACAGAGGCTTACACAAC-3^′^, and *RNF128* reverse: 5^′^-TCAGTCTTACCTCTTTGCCACTAG-3^′^.

**Table 2:** Description of the region amplified by the primers.

Name	GenBank accession	Location	Exon	Amplicon length	Annealing
	no. position		count	(bp)	temperature (Ta; °)
RNF34	NC_037344.1	Chromosome 17	8	329	60
RNF128	NC_037357.1	Chromosome X	9	526	59

**Figure 1:**
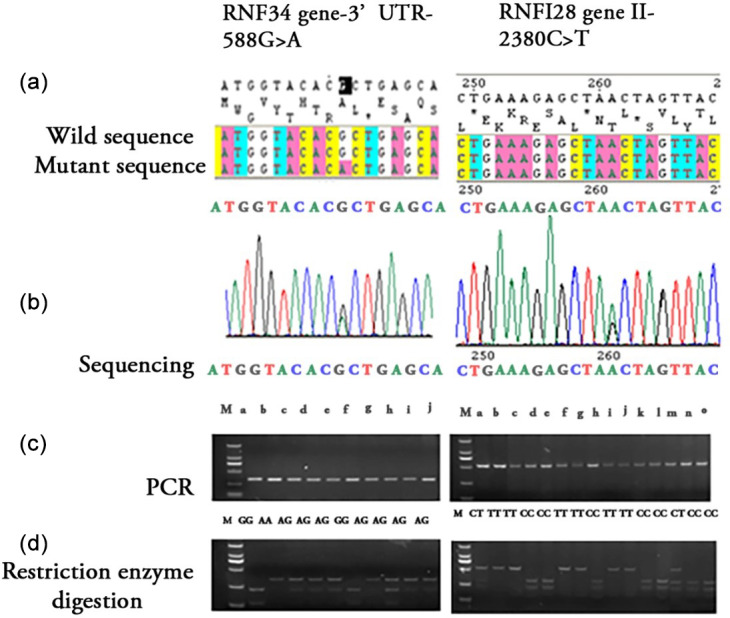
Identification of bovine *RNF34* gene 3^′^ UTR-588G > A and *RNF128* gene I1-2380C > T by sequencing and restriction enzyme digestion analysis of PCR products. **(a)** Wild-type and mutant sequences of the *RNF34* 3^′^ UTR-588G > A and *RNF128* I1-2380C > T genes. The sequences shown highlight the nucleotide changes from G to A (*RNF34*) and C to T (*RNF128*). **(b)** Sequencing chromatograms showing the SNP locations for *RNF34* 3^′^UTR-588G > A and RNF128 I1-2380C > T. The chromatograms illustrate the nucleotide variations at positions 588 and 2380, respectively. **(c)** PCR amplification results displaying the genotypes for the *RNF34* and *RNF128* SNPs. Lanes are labeled according to the genotype of the individual animals, with M indicating the DNA marker. **(d)** Restriction enzyme digestion analysis of the PCR products for *RNF34* and *RNF128*. The digested products are shown for each genotype, demonstrating the presence or absence of the restriction site corresponding to the SNP.

A reaction mixture of total 20 µ L volume was prepared for a polymerase chain reaction: bovine genomic DNA (100 ng), each primer (10 pmol L^−1^), deoxynucleotide triphosphates (dNTP) (200 µ mol L^−1^ of each), MgCl2 (1.5 mmol L^−1^), 2.5 µ L buffer ( 10× concentrate: 200 mmol L^−1^ Tris-HCl, pH 8.4, 500 mmol L^−1^ KCl), and 1.0 unit of Taq DNA polymerase. The following cycling conditions were given for PCR amplification (Xiao et al., 2016): 95 °C for 5 min (initial denaturation) followed by 30 cycles of 95 °C for 30 s (denaturation), 60 °C for 30 s (annealing), and 72 °C for 40 s (extension), with a final extension step at 72 °C for 10 min, as shown in Fig. 1.

#### 2.2.6 Genomic DNA extraction

Genomic DNA was extracted from the collected blood samples using a Tiangen DNA extraction kit (Tiangen, Beijing, China) according to the manufacturer's protocol. Briefly, 1 mL of whole blood was mixed with a lysis buffer and proteinase K, which was followed by incubation at 56 °C until complete lysis. The samples were then subjected to ethanol precipitation, and the DNA was purified using a silica-based spin column. Concentration and quality of the DNA were determined using a NanoDrop (Thermo Fisher Scientific, USA) spectrophotometer and agarose gel electrophoresis. The DNA extracts were then kept frozen at - 20 °C until further use.

#### 2.2.7 Development of SNP library

A SNP library was created for the analysis of polymorphisms that exist within two putative target genes, *RNF34* and *RNF128*. The target regions, the 3^′^ UTR of *RNF34* and intron 1 of *RNF128* were amplified with specific primers designed on reference sequences from NCBI. All sequences were confirmed by Sanger sequencing of purified PCR products using a QIAquick PCR Purification Kit (Qiagen, Hilden, Germany). The sequencing data were analyzed using the BioEdit software to search for single-nucleotide polymorphisms within the target regions. The identified SNP were subjected for association analysis with carcass and meat quality traits.

#### 2.2.8 Selection of gene regions

This study focuses on the 3^′^ UTR of *RNF34* and intron 1 of *RNF128* due to their potential to harbor SNPs that affect gene expression (Liu et al., 2008a; Wang and Deng, 2011a). The 3^′^ UTR regulates mRNA stability and protein levels, directly influencing traits like carcass and meat quality (Schartner et al., 2009a; Petroski and Deshaies, 2005a). Focusing on these regions allowed for a more efficient and cost-effective investigation of genetic variations linked to these traits.

#### 2.2.9 SNP detection and genotyping

Two polymorphisms in *RNF34* and *RNF128* genes were identified through the sequencing and confirmed through REA (restriction endonuclease analysis) using enzymes as restriction endonuclease (Bpu1102 and Spe I, New England Biolabs, MA, USA). Bpu1102 was used to distinguish the genotypes of the PCR products of the *RNF34* gene while Spe I used for *RNF128* gene. Restriction digestion of the amplicons of the SNPs of two genes was conducted at 37 °C for 6 h – 18 µ L, final volumes with PCR product (8 µ L), enzyme buffer (1.8 µ L), and restriction enzyme (0.4 µ L). The products of PCR digestion of both genes were resolved and visualized on 3 % agarose gels to distinguish the bands representing three different genotypes (Tian et al., 2013; Xiao et al., 2016; Li et al., 2017).

#### 2.2.10 Statistical analysis

Allelic and genotypic frequencies for polymorphism of each gene were determined based on its information content to understand the diversity in the studied population. Genotype frequencies were calculated and analyzed by significance testing. The genotypic effects of the *RNF34* gene and *RNF128* gene were determined using the general linear model (GLM) and one-way ANOVA using SPSS (version 13.0) (Korkmaz-Ağaoğlu et al., 2019).

## 3 Results

### 3.1 PCR amplification

In total, genomic DNA from 255 Simmental-cross steers was amplified using gene-specific primers targeting the *RNF34* and *RNF128* genes. The PCR amplification products observed (intron 1) corresponded to the expected target fragments with good specificity. The PCR products were directly analyzed by restriction enzyme digestion and sequencing reactions.

The decision to investigate the 3^′^ UTR SNP of *RNF34*, despite the intron 1 of *RNF128* yielding good specificity, was based on the distinct functional roles these regions play in gene expression and regulation. The 3^′^ UTR (untranslated region) of a gene is critical for the regulation of mRNA stability, localization, and translation efficiency.

Although selected for the specificity of PCR amplification (intron 1 region in *RNF128*) or potential to influence gene expression at post-transcriptional level (3^′^ UTR, both in *RNF34*), these regions happen within highly polymorphic genes and hence detect only a minor portion of genetic diversity. This is a region that was selected due to previous analysis implicating SNPs in the 3^′^ UTR as likely having biological significance, which would be an appropriate candidate gene region for exploring genetic impacts on economically important phenotypes.

### 3.2 Restriction endonuclease analysis and sequencing different genotypes

The PCR amplified products of *RNF34* and *RNF128* were subjected to SNP detection, genotyping, moreover, the two polymorphisms in both genes observed upon DNA sequencing using Sango tools (Shanghai, China). A single-nucleotide polymorphism site at nucleotide 588 in the *RNF34* gene is an essential mediator of transcriptional and post-translational regulation (Fig. 1). PCR products from 255 samples were digested with the Bpu1102 restriction enzyme and resolved on a 3 % agarose gel for genotyping identification. A single band indicated a homozygous AA genotype, two bands indicated a homozygous GG genotype, and three bands indicated a heterozygous AG genotype, as shown in Fig. 1. Among the 255 Simmental-cross steers, the allele frequency for G at the 3^′^ UTR-G588A polymorphism site is 0.5882, whereas the frequency for allele A is 0.4118. Multi-comparison statistical analyses showed no statistically significant difference ( P>0.05 ) in genotype distributions (Table 3). Using fragment length for *RNF128*, the sequencing results indicated that there was an SNP of I1-2380C > T in the first intron region. Following Spe I restriction, the following frequencies were found for the 213 samples: TT genotype of 0.5305, TC genotype of 0.0657, CC genotype of 0.4038, T allele of 0.5634, and C allele of 0.4366.

**Table 3:** Genotypic frequencies of SNPs in *RNF128* and *RNF34* genes of different cattle populations.

Population	*RNF34* gene 3^′^ UTR-588G > A			*RNF128* gene I1-2380C > T		
	Number	Allele frequency	Genotype frequency	Number	Allele frequency	Genotype frequency
Chinese Simmental cattle	255	G (0.5882) A (0.4118)	GG (0.2823) GA (0.6118) AA (0.1059)	213	T (0.5634) C (0.4366)	TT (0.5305) TC (0.0657) CC (0.4038)

Of the 255 samples, 213 were successfully genotyped and included in the statistical analysis. Approximately 40 samples were excluded due to issues such as poor DNA quality, insufficient quantity after extraction, or incomplete data, which prevented successful PCR amplification and sequencing. These exclusions were necessary to ensure the accuracy and reliability of the results presented.

### 3.3 Association analyses of *RNF34* and *RNF128* polymorphisms with carcass and meat quality traits

One-way analysis of variance (ANOVA) was employed to explore the link between *RNF34* polymorphisms and carcass traits of selected animals. Statistical analyses revealed that *RNF34* 3^′^ UTR (c. + 588G > A) had a significant association with the carcass and meat quality traits, including tare weight, kidney weight, testis weight, and fat color score ( P<0.05 ), as shown in Table 4.

**Table 4:** *RNF34* 3^′^ UTR (c. + 588G > A) association with the carcass and meat quality traits.

Trait	*RNF34* genotypes (3^′^UTR-588G > A)								
	AA			AG			GG		
	N	LSM	SE	N	LSM	SE	N	LSM	SE
GW (kg)	27	519	58.48	156	511.76	56.61	72	501.04	55.4
CW (kg)	27	275.85	35.15	156	270.81	35.66	72	265.22	33.15
DP (%)	27	53.09	2.02	156	52.83	2.27	72	52.88	1.62
NWB (kg)	27	19.78	1.97	156	19.81	3.41	72	19.69	2.71
HW (kg)	27	23.9	2.2	156	23.72	2.32	72	23.25	2.39
FW (kg)	27	6.21	0.69	156	6.06	0.72	72	6.03	0.65
WH (kg)	27	2.89	0.4	156	2.81	0.38	72	2.8	0.34
PW (kg)	27	40.93^a^	4.35	156	39.92^a^	4.62	72	38.55	4.53
LI (kg)	27	0.61	0.12	156	0.62	0.15	72	0.61	0.15
SI (kg)	27	9.18	1.38	156	9.13	1.01	72	9.12	1.05
RW (kg)	27	7.18	0.85	156	7.21	0.8	72	7.09	0.75
OW (kg)	27	3.63	0.48	156	3.64	0.53	72	3.74	0.5
HW (kg)	27	1.97	0.26	156	1.99	0.27	72	1.96	0.3
LW (kg)	27	6.47	0.55	156	6.57	0.76	72	6.48	0.63
LT (kg)	27	3.34	0.34	156	3.37	0.39	72	3.28	0.42
KW (kg)	27	1.31^a^	0.15	156	1.26^ab^	0.19	72	1.23^b^	0.15
SW (kg)	27	0.85	0.18	156	0.92	0.18	72	0.91	0.18
BP (kg)	27	0.41	0.06	156	0.41	0.06	72	0.39	0.05
TW (kg)	27	0.68^ab^	0.18	156	0.72^a^	0.14	72	0.67^b^	0.16
OT (kg)	27	1.48	0.2	156	1.47	0.21	72	1.43	0.22
MFW (kg)	27	4.94	1.52	156	4.92	1.12	72	4.76	0.88
SO (kg)	27	2.72	1.35	156	2.57	0.86	72	2.35	0.89
KFW (kg)	27	6.12	2.26	156	6.38	2.02	72	6.15	2.12
GF (kg)	27	0.82	0.37	156	0.81	0.36	72	0.78	0.32
CL (cm)	27	136.04	6.8	156	137	7.45	72	136.51	7.56
CD (cm)	27	63.96	3.72	156	64.06	3.24	72	63.77	2.95
CCD (cm)	27	65.89	4.13	156	64.82	3.64	72	64.39	3.32
HLC (cm)	27	49.76	2.64	156	49.33	2.95	72	49.47	4.95
HLW (cm)	27	45.02	2.25	156	44.45	2.75	72	44.87	2.89
HLL (cm)	27	78.96	2.86	156	78.11	3.06	72	77.85	2.77
TMT (cm)	27	18.06	1.73	156	18.22	1.61	72	17.97	2.02
TL (cm)	27	7.15	0.69	156	7.36	0.77	72	7.28	0.79
BFT (cm)	27	1.33	0.55	156	1.3	0.44	72	1.37	0.49
FCR (%)	27	63.85	13.84	156	62.21	9.95	72	61.46	11
SpH	27	5.9	0.36	156	5.97	0.27	72	5.99	0.35
PpH	27	5.37^ab^	0.3	156	5.39^a^	0.27	72	5.48^b^	0.33
SF (kg)	27	4.56	1.47	156	4.62	1.45	72	4.39	1.41
MBS	27	5.11	0.7	156	5.09	0.69	72	5.21	0.77
EMA	27	83.56	11.01	156	83.17	12.36	72	84.36	12.83
FCC	27	5.96	1.13	156	5.95	1.02	72	5.92	0.96
FCI	27	84.34	8.7	156	82.93	8.53	72	83.57	6.78
FCS	27	2.15^ab^	0.72	156	2.24^a^	0.84	72	2.50^b^	0.82

Values within a row that share the same letter (uppercase or lowercase) are not significantly different from each other ( P>0.05 ). Different letters indicate statistically significant differences ( P<0.05 ).

**Table 5:** Association of *RNF128* SNPs with carcass and meat quality traits in Simmental-cross steers.

Trait	*RNF128* genotypes (I1-2380C > T)								
	TT			TC			CC		
	N	LSM	SE	N	LSM	SE	N	LSM	SE
GW (kg)	113	507.31^a^	56.85	14	542.21	36.79	86	499.65^A^	53.57
CW (kg)	113	268.82^ab^	34.88	14	286.64^a^	25.13	86	265.23^b^	34.26
DP (%)	113	52.92	2.05	14	52.82	2.04	86	52.98	1.79
NWB (kg)	113	19.88^ab^	3.34	14	21.14^a^	2.01	86	19.37^b^	2.93
HW (kg)	113	23.47	2.46	14	24.43	1.67	86	23.31	2.15
FW (kg)	113	6.07^a^	0.71	14	6.49	0.6	86	5.95^A^	0.67
WH (kg)	113	2.80^A^	0.38	14	3.13	0.47	86	2.77^A^	0.33
PW (kg)	113	39.54	4.84	14	40.71	3.9	86	38.75	4.31
LI (kg)	113	0.6	0.12	14	0.6	0.14	86	0.62	0.17
SI (kg)	113	9.13	1.09	14	9.46	1.18	86	9.09	0.99
RW (kg)	113	7.13^a^	0.81	14	7.66	0.68	86	7.13^a^	0.77
OW (kg)	113	3.64^a^	0.51	14	3.98	0.58	86	3.59^A^	0.48
HW (kg)	113	1.99	0.31	14	2.01	0.15	86	1.94	0.26
LW (kg)	113	6.56	0.71	14	6.55	0.59	86	6.44	0.65
LT (kg)	113	3.31^A^	0.4	14	3.64	0.4	86	3.30^A^	0.39
KW (kg)	113	1.26	0.2	14	1.29	0.15	86	1.23	0.16
SW (kg)	113	0.91	0.18	14	0.89	0.16	86	0.9	0.18
BP (kg)	113	0.40^ab^	0.06	14	0.43^a^	0.07	86	0.39^b^	0.06
TW (kg)	113	0.7	0.14	14	0.75	0.18	86	0.71	0.16
OT (kg)	113	1.43^a^	0.21	14	1.57	0.14	86	1.44^a^	0.19
MFW (kg)	113	4.88^ab^	1.13	14	5.43^a^	1.01	86	4.71^b^	0.99
SO (kg)	113	2.51	0.85	14	2.67	0.97	86	2.45	0.95
KFW (kg)	113	6.22	2.12	14	5.87	1.84	86	6.23	1.93
GF (kg)	113	0.8	0.33	14	0.86	0.41	86	0.81	0.39
CL (cm)	113	136.11	7.92	14	138.46	4.15	86	136.37	7.44
CD (cm)	113	63.74^a^	3.33	14	65.75	2.87	86	63.52^a^	2.78
CCD (cm)	113	64.62^a^	3.85	14	66.71	2.74	86	64.15^a^	2.9
HLC (cm)	113	49.22^a^	3.14	14	51.32^b^	2.85	86	49.60^ab^	4.29
HLW (cm)	113	44.5	2.43	14	45.39	2.18	86	44.64	3.11
HLL (cm)	113	77.87^A^	3.03	14	80.61	2.98	86	77.76^A^	2.74
TMT (cm)	113	18.02^A^	1.69	14	19.4	1.04	86	18.07^A^	1.89
TL (cm)	113	7.33^ab^	0.79	14	7.71^a^	0.68	86	7.17^b^	0.7
BFT (cm)	113	1.3	0.46	14	1.39	0.37	86	1.32	0.46
FCR (%)	113	62.16	11.46	14	65	11.44	86	60.68	8.95
SpH	113	6.03^A^	0.36	14	5.99^AB^	0.32	86	5.89^B^	0.35
PpH	113	5.43	0.34	14	5.43	0.22	86	5.42	0.25
SF (kg)	113	4.48	1.41	14	4.52	1.76	86	4.62	1.44
MBS	113	5.14	0.72	14	5.36	0.75	86	5.14	0.71
EMA	113	83.6	12.48	14	84	9.94	86	83.99	12.25
FCC	113	6.06	1	14	5.93	0.92	86	5.79	1
FCI	113	84.02	7.02	14	81.8	8.49	86	82.78	9.13
FCS	113	2.35	0.86	14	2.5	0.65	86	2.35	0.79

Values within a row that share the same letter (uppercase or lowercase) are not significantly different from each other ( P>0.05 ). Different letters indicate statistically significant differences ( P<0.05 ). GW: gross weight; CW: carcass weight; DP: dressing percentage; NWB: net weight of bone; HW: head weight; FW: forepaw weight; WH: weight heels; LI: large intestine; SI: small intestine; RW: rumen weight; OW: omasum weight; HW: heart weight; LW: liver weight; LT: lung, trachea; KW: kidney weight; SW: spleen weight; BP: bull penis; TW: testes weight; OT: ox tail; MFW: mesenteric fat weight; SO: stomach oil; KFW: kidney fat weight; GF: genital fat; CL: carcass length; CD: carcass depth; CCD: carcass chest depth; HLC: hind leg circumference; HLW: hind leg width; HLL: hind leg length; TMT: thigh meat thickness; TL: thickness of loin; BFT: back fat thickness; FCR: fat coverage rate of carcass; SpH: slaughter pH; PpH: pH after acid exhausted; SF: shearing force; MBS: marbling score; EMA: eye muscle area; FCC: flesh color (color card); FCI: flesh (flesh-colored instrument); FCS: fat color score; LSM: least-squares mean; SE: standard error of mean.

One-way ANOVA and the least significant difference (LSD) test analyzed associations of the *RNF128* gene polymorphisms with carcass traits to allow for multiple comparisons to be conducted concerning production traits. As illustrated in Table 5, the differences were significant between the different genotypes, involving forepaw weight, dressed weight, carcass depth, the thickness of waist flesh, carcass brisket depth, slaughter pH ( P<0.05 ), lungs, trachea, and hind leg length ( P<0.01 ).

Given the large number of characteristics analyzed, which includes correlated traits, there is potential for inflated type I error rates. To address this, a Bonferroni correction was applied to adjust the P values for multiple testing, ensuring that the reported significant associations are robust. Alternatively, the analysis was focused on a subset of essential characteristics most relevant to meat and carcass quality, reducing the potential for spurious findings.

## 4 Discussion

Meat quality has great commercial importance for the animal husbandry industry. This industry heavily relies on the genetic background of the animals and their management, nutrition, and meat processing (Dong et al., 2019) although previous studies mainly focused on *RNF34* and *RNF128* were associated with cell differentiation and apoptosis (Verdin, 2010).

The observed differences in meat pH associated with the *RNF34* and *RNF128* SNPs may have important implications for meat quality. A lower pH in the animals with *RNF34* 3^′^ UTR-588. An allele may suggest a more rapid post mortem glycolysis, resulting in a faster decrease in its pH. This in turn can impact meat color, tenderness, and shelf life, which are salient factors for consumer acceptability. On the other hand, although not significant, pH increased by a slight level with I1-2380T allele in *RNF128* could indicate a reduced rate for glycolysis that is another aspect that leads to differences on meat texture and water-holding capacity (Huff-Lonergan and Lonergan, 2005). This work revealed the possible influence of RNF34 and *RNF128* on post mortem muscle metabolism, which may provide some new insight into meat quality traits, but more investigation is needed for use in marker-assisted selection.

Results show that *RNF34* and *RNF128* polymorphisms significantly correlates with certain meat quality traits in the study. These associations may be explained by *RNF34*’s regulatory effects on mRNA stability influencing muscle and fat tissue development as well as its role in T-cell function through transcriptional regulation. However, the effects of *RNF34* on post-transcriptional regulation indicate that it might regulate protein synthesis in muscle cells and thus exert its influence via medication with these mRNA modifications.

*RNF34* transcripts are highly enriched in brilliant cresyl blue (BCB) oocytes, suggesting that *RNF34* may be involved in oocyte apoptosis (Wraith et al., 2009; Petroski and Deshaies, 2005b). And *RNF34* plays a crucial role in the regulation of NOD1, *RNF34*, NF- κ B pathways, which supports the idea that *RNF34* is a negative regulator of the NOD1 pathway through direct interaction and ubiquitination of NOD1 (Skog et al., 2004; van Wijk et al., 2009). *RNF128*, an E3 ubiquitin-protein ligase, is an important gatekeeper of T cell unresponsiveness and uses a special single transmembrane protein with a split-function motif (Gavali et al., 2021). It may have a prominent role in CD4 T cell functions, including its survival, activation, and differentiation, where GRAIL acts in a well-characterized manner as it negatively regulates the responsiveness of T cell receptors and production of cytokine (Lett et al., 2004; Lineberry et al., 2008). However, new important roles of *RNF34* and *RNF128* are found in the present research.

The significant associations observed between *RNF34* and *RNF128* polymorphisms and meat quality traits in this study are consistent with findings from previous QTL and GWAS studies, which have highlighted the potential importance of these genes in livestock production. Although *RNF34* and *RNF128* are not traditionally associated with meat quality traits, their involvement in broader biological processes such as protein turnover, energy metabolism, and immune response provides a plausible link to the observed phenotypic variations (Petroski and Deshaies, 2005a; Schartner et al., 2009a). These results suggest that *RNF34* and *RNF128* may serve as novel markers for meat quality in cattle, warranting further investigation in larger and more genetically uniform populations to validate these findings.

In this study, SNPs of *RNF34* and *RNF128* are found to play important roles in meat quality traits and growth traits. SNP of *RNF34* 3^′^ UTR-588 G > A suggested that the AA genotype is significantly associated with testis weight and fat color score (Yu et al., 2017). The AG genotype is considerably associated with the weight of the kidney. Furthermore, the average production data for cattle of genotype AA are lower than those for cattle with AG or GG in pH after acid exhaustion and fat color score. The *RNF128* gene I1-2380C > T shows that different genotypes significantly influence the carcass traits. There is a noticeable association between the TT genotype and the carcass weight, net weight of bone, bullwhip weight, mesenteric fat weight, and thickness of waist flesh. The TC genotype is significantly associated with slaughter pH. The CC genotype is significantly linked with hind leg circumference. The LSM of the carcass weight, net weight of bone, bullwhip weight, mesenteric fat weight, thickness of waist flesh, and hind leg circumference for the TC genotype higher than that of the TT or CC genotypes. The LSM of slaughter pH was calculated, and it is higher for the TT genotype than that for the TC or CC genotypes. So, we can select the excellent meat traits by genotypes of *RNF34* and *RNF128*. Thus, this study supports the development of a novel theory about the cultivation of excellent beef using molecular biology techniques. Our results confirmed promising findings showing some previously mentioned associations but divergence from some studies, highlighting their limitations as further experimentations and required refinements for further validation of these associations.

## 5 Conclusion

This study provides evidence supporting the involvement of *RNF34* and *RNF128* in carcass and meat quality traits in Chinese Simmental-cross steers. The selection of these genes was informed by their known roles in ubiquitination and immune regulation as well as by QTL and GWAS studies that suggest a broader relevance to growth and meat quality traits in livestock. While the direct functions of these genes do not imply a causal link, their indirect effects on metabolic and cellular processes could influence economically important traits in cattle. Future research should aim to validate these findings in larger populations and explore the underlying mechanisms through which *RNF34* and *RNF128* may affect meat quality.

## Data Availability

The data presented in this study are available from the corresponding authors upon reasonable request.
